# Post-ruminal effects of rumen-protected methionine supplementation with low protein diet using long-term simulation and in vitro digestibility technique

**DOI:** 10.1186/s13568-018-0566-7

**Published:** 2018-03-09

**Authors:** Imtiaz Hussain Raja Abbasi, Farzana Abbasi, Mohamed E. Abd El-Hack, Ayman A. Swelum, Junhu Yao, Yangchun Cao

**Affiliations:** 1 College of Animal Science and Technology, Northwest A&F University, Yangling, 712100 Shaanxi People’s Republic of China; 2 School of Life Science and Engineering, Southwest University of Science and Technology, Mianyang, 621010 Sichuan People’s Republic of China; 3 Department of Poultry, Faculty of Agriculture, Zagazig University, Zagazig, 44511 Egypt; 4 Department of Animal Production, College of Food and Agriculture Sciences, King Saud University, P.O. Box 2460, Riyadh, 11451 Saudi Arabia

**Keywords:** In-vitro digestibility, Methionine, Protein, Post-ruminal, Rusitec

## Abstract

Microbial degradation in the rumen and dietary availability of methionine amino acid have been reported as limiting in dairy ruminants. The aim of the present study was to examine the post-ruminal effects of feeding ruminants different concentrations of rumen-protected methionine (RPM) in low crude protein diets using the long-term rumen simulation method (Rusitec) followed by in vitro abomasum and ileum digestibility technique. The experiment contained four treatment groups: (1) high protein, without RPM supplementation (HP); (2) low protein, without RPM supplementation (LP); (3) low protein supplementation with low RPM (LPLM); and (4) low protein supplementation with high RPM (LPHM) mixed per 20 ± 0.04 g basal diet in every fermenter. The results showed that the LPLM and LPHM groups had significantly higher disappearance of crude protein and neutral detergent fiber in the abomasum and ileum than the HP treatment (*P *< 0.05) and were the same as the LP group (*P *> 0.05). The proportions of short-chain fatty acids and total volatile fatty acids in the abomasum and ileum were the same between the LPHM and HP groups (*P *> 0.05); however, the LPLM group was found to be significantly (*P* < 0.05) lower than the HP group and similar to the LP group (*P *> 0.05). Rusitec pH before or after changing feed bags and daily ammonia nitrogen production in the abomasum and ileum were non significantly (*P* > 0.05) different among all groups. In conclusion, RPM supplementation with low crude protein diets promoted post-ruminal digestibility and production of volatile fatty acids.

## Introduction

Proteins, specifically amino acids profiles, are the most limiting nutrient for milk production in ruminants with high genetic merit. To solve this problem, dietary supplementation with rumen-protected amino acids (RPAA) is a suitable approach (Elek et al. 2008; Osorio et al. 2013). The main objective of any nutritional program is to formulate diets that match the nutritional requirements of the animal to their age and stage. Moreover, it is important to promote the production of short-chain fatty acids (SCFAs), which are known as key bacterial metabolites, for synthesis by dietary carbohydrate fermentation in the gastrointestinal tract of animals (Tan et al. 2014). In ruminants, 70–80% of energy for the rumen epithelia and 50–70% for the overall energy requirements are provided by SCFAs and a variety of physiological functions are maintained by the rumen epithelium (Bergman 1990; Tufarelli et al. 2009). Furthermore, digestibility of nutrients is expressed as the amount that is absorbed by the body of an animal and the utilization of those nutrients for growth, reproduction, and other functions. Data suggest that dry matter intake (DMI) and digestibility of nutrients are major factors influencing nutrient utilization in dairy cattle, yet in lactating dairy cows, DMI is driven by the production of milk but sometimes limited by physical fill and metabolic effects (Allen 2000). Feed is a rich source of proteins; however, it is hard to digest and animals cannot maintain the amino acids (AA) balance between the high production of milk and supplementation of free AA and the balance of essential AA, which limits the key methyl donor, methionine, in dairy ruminants. In this situation, RPAA supplementation is a common method to counteract AA deficiency in dairy cow diets (Abbasi et al. 2018). The AA products are produced from ruminal fermentation and aim to enable maximum flow of AA concentrations in the small intestine. Supplementation of RPAA can increase the flow of AA in the duodenum and absorption also significantly promotes production performance in dairy cattle (Lara et al. 2006). Methionine is a sulfur AA that plays a major role in many pathways, e.g., synthesis of phospholipids, carnitine, creatine, and polyamines, and a source of the methyl donor S-adenosyl methionine (Abbasi et al. 2018). Moreover, methionine is also utilized for protein synthesis and can donate methyl groups for a different reaction and to deliver sulfur groups from the synthesis of cysteine. Therefore, it is important to balance and maintain all these methionine-dependent key functions in dairy ruminants via the supply of additional methionine into a protected form (i.e., rumen-protected methionine, RPM) for post-ruminal maximum absorption, particularly in the small intestine. In many studies, methionine has been reported as being one of the most important AA required for growth of small and large ruminants (Wiese et al. 2003). It is involved in many vital metabolic processes including serving as a glycogenic AA (Brosnan 2003), as a precursor to cysteine, and as a constituent of various vital proteins, e.g., glutathione peroxidase and apolipoprotein B-100 (Brosnan 2003). Additionally, methionine also plays a direct role in low density lipoprotein synthesis in bovines and decreases plasma ketones during early lactation (Girard and Matte 2005; Abbasi et al. 2017). Elevating blood plasma methionine levels might promote better liver function, increase antioxidant levels, decrease inflammation, and improve oxidative stress capacity (Osorio et al. 2013). If maximum degradation of methionine in the rumen or free supply occurs, then this causes a limitation of methionine in dairy ruminants. Therefore, a negative methyl donor balance is also a significant challenge for peripheral dairy cows. Several techniques have been developed to ensure the proper supply of methionine including encapsulation or matrix protection of methionine (Patton 2010) into the diets of ruminants to balance energy by increasing glucose production, promote hepatic oxidation of AA, or directly endorse protein synthesis.

Our hypothesis was that rumen-protected DL-methionine was degraded more slowly by rumen microorganisms, moderately in the abomasum, and fully in the small intestine of the ruminant, thereby providing a steadier supply of methionine to the small intestine. Thus, the main purpose of the present study was to examine the effects of feeding different levels of rumen-protected DL-methionine supplementation with low crude protein (CP) on post-ruminal fermentation, disappearance, production of volatile fatty acids, and ammonia nitrogen (NH_3_-N) production.

## Materials and methods

### Animal care

Care of animals and all experimental procedures in the study were permitted by the Institutional Animal Care and Use Committee (IACUC) of the College of Animal Science and Technology of the Northwest A&F University (Yangling, Shaanxi, PR China).

### Simulation apparatus and test product

The experiment was performed by the application of the rumen simulation technique (Rusitec; Sanshin, Tokyo, Japan) as designated by Kajikawa et al. (2003) and in vitro digestibility was undertaken based on the modified technique by Boisen and Fernhndez (1995). The test product was manufactured by Mepron^®^ (Degussa Corporation, Germany) as a sample of a surface-coated, carbohydrate-protected product. The pellets contained a core of methionine that was coated with numerous fine layers of ethyl-cellulose and stearic acid. The final product was composed of a minimum 85% DL-methionine, approximately 8.5% carbohydrates in non-structural form, 3.5% neutral detergent fiber (NDF), 1.5% ash, 1.0% moisture, and 0.5% crude fat.

### Inoculum donor and experimental diets

Four healthy Xinong Saanen goats [average initial body weight (55.6 ± 1.40 kg)], were used for inoculum. Rumen content was collected through the ruminal fistula after 4:00 h morning feeding into pre-heated at 39 °C flasks and transferred to the laboratory. The Rusitec experiment designed randomly and conducted over two independents 15 days incubation periods with 7 days for adaption and 8 days for samples collection. The four groups were as follows: (1) high protein without RPM (HP, 163.39 g/kg CP), (2) low protein without RPM (LP, 146.33 g/kg CP), (3) low protein (141.80 g/kg CP) supplemented with low concentration of RPM (0.11 g/kg DM) (LPLM), and (4) low protein (143.30 g/kg CP) supplemented with high concentration of RPM (0.81 g/kg DM) (LPHM). HP diet formulated as a positive control group based on NRC (2001) recommendation and LP was designed as a negative control group shown in Table 1. Diets were formulated by using the Cornell-Penn-Miner Dairy (CPM-Dairy, Version 3.0.8.1) Software and balanced according to NRC (2001) requirements. In vitro, one step abomasum and ileum digestibility were followed by the modified procedure of Boisen and Fernhndez (1995), by utilizing undigested Rusitec residue for abomasum digestibility, and for further ileum digestibility, the abomasum residue was utilized.

**Table 1:** Ingredients and chemical composition of the experimental diets fed by donor animals and supplied daily to each fermenter (DM basis, g/day)

Items	Treatments			
	HP	LP	LPLM	LPHM
Ingredients supply (g/day)				
Corn silage	3.65	3.87	3.87	3.87
Alfalfa hay	2.03	0.78	0.78	0.78
Wheat straw	3.06	4.28	4.27	4.27
Ground corn	3.25	4.33	4.33	4.33
Wheat bran	1.56	0.94	0.94	0.93
Soybean meal	1.41	2.01	2.01	2.01
Cottonseed meal	1.92	0.76	0.76	0.76
Corn germ meal	2.31	2.11	2.11	2.11
Limestone	0.21	0.18	0.18	0.18
Dicalcium phosphate	0.00	0.10	0.10	0.10
NaCl	0.21	0.24	0.24	0.24
NaHCO_3_	0.04	0.04	0.04	0.04
Premix^a^	0.36	0.36	0.36	0.36
RPM	0.0000	0.0000	0.0021	0.0200
Chemical composition				
Dry matter (g/day)	9.03	8.90	8.92	8.94
Crude protein (g/day)	1.63	1.42	1.42	1.43
aNeutral detergent fiber (g/day)	4.71	4.72	4.71	4.70
Acid detergent fiber (g/day)	2.12	2.11	2.05	2.12
Ether extract (g/day)	0.29	0.28	0.28	0.28
Gross energy (MJ/day)	0.16	0.16	0.16	0.17

^a^Premix (contains): 9.80 mg Zn, 10.5 mg Cu, 12.00 mg Mn, 0.32 mg I, 0.15 mg Se, 0.11 mg Co, 500 IU vitamin D3, 40 IU vitamin E, and 2500 IU vitamin A, per kilogram of TMR on DM basis

### Experimental technique and sampling

After collection, the inoculum was strained through four layers of cheese cloth maintained under anaerobic conditions. On day 1, each Rusitec fermenter was filled with 350 mL strained liquid inoculum and an equivalent volume of McDougall’s buffer solution based on the procedure described by McDougall (1948). One nylon bag containing 70 ± 0.05 g of solid rumen digesta (wet weight) and one nylon bag containing 20 ± 0.04 g of the experimental diet comprising roughage and concentrate (45:55 DM basis), which was ground and then sieved through 4 and 2 mm sieves, were added to the fermenters. After 24 h, the bag containing the solid inoculum was swapped with a new feed bag in each fermenter. The next day, the old bag was replaced with a new one; therefore, each experimental bag was incubated for 48 h. Freshly prepared artificial saliva (McDougall’s buffer) was continuously imbued into each Rusitec fermenter through a pump at a flow rate of 626 mL/day following the methods described by Kajikawa et al. (2003). After completion of the adaptation period, on day 8, 9, and 10, the pH of each fermenter was measured before exchanging the feed bag. On day 11 and 12 after substituting the feed bag from each fermenter, 6 mL of fluid was used to measure pH at 0, 3, 6, 9, 12, 18, 21, and 24 h, respectively.

On days 13, 14, and 15, one feed bag (containing residue) from every Rusitec fermenter was collected, washed with 100 mL of McDougall’s buffer followed by a cold rinse cycle for 10 min with cold water and dried in an air forced oven for 24 h at 65 °C for further in vitro abomasum digestibility.

Disappearance of DM and other indexes were calculated as (g/kg DM):

$${\text{Disappearance}}\, = \,\left[ {\left( {{\text{W}}_{ 3} {-}{\text{W}}_{ 4} } \right)/{\text{W}}_{ 3} } \right]\, \times \, 1000$$

where, W_3_ is the % DM in the feed sample [(W_1 _− W) × DM%]; W_4_ is the residue DM weight [W_2 _− W] × 100; W is the weight of the empty bag; W_1_ is the weight of the bag with feed sample before incubation; and W_2_ is the weight of the bag with residue after incubation.

### In vitro digestibility (abomasum and ileum)

Boisen and Fernhndez (1995) introduced a three-step procedure which was further modified into two-steps by using incubation of the samples with pepsin enzyme, and then further incubation carried out with small intestinal fluid for 18 h. The small intestine fluid was collected and prepared according to Liang (2013). After incubation of experimental feed into Rusitec, the undigested residue was dried at 65 °C and weighed 2.50 ± 0.04 g DM basis into 100 mL bottles (8 replicates per treatment) for in vitro abomasum digestibility with 6 h incubation by automatic incubactor and shaker. Furthermore, after incubation at abomasum, the contents were filtered through two layers of cheese cloth, the residue was dried at 65 °C and weighted 0.60 ± 0.05 g DM basis pour into 100 mL bottles with 10 mL of fluid from abomasum and 30 mL of artificial saliva were added with same replication for in vitro ileum digestibility. Incubation was carried out for 18 h in an automatic incubactor and shaker, contents were filtered fluid and undigested residue (abomasum and ileum) were collected for further analysis.

### Chemical analysis

All the dried feed, residues from post-ruminal (abomasum and ileum) incubation were mixed thoroughly following experimental groups with treatment and ground to pass a 1-mm screen in a Wiley mill for use in chemical analyses. The samples were analyzed for dry matter (DM) content (method 934.01; AOAC 2004), ether extract (EE, method 920.30; AOAC International 2004) and crude protein (CP) (method 988.05; AOAC International 2004). Neutral detergent fiber (aNDF) and acid detergent fiber (ADF) were resolute by Van Soest et al. (1991) method, using an ANKOM^200^ fiber analyzer (Ankom Technology, USA). Using the reagents and filter bags recommended by the manufacturer Ankom Technology, USA. Further, at analysis was conducted with a heat stable α-amylase with sodium sulfite and expressed as inclusive residual ash. When collecting Rusitec fermenter fluid, pH was measured quickly by portable pH meter (Orion 230 A-plus, Thermo Scientific, Beverly, MA, USA). The concentrations of volatile fatty acids (VFA) was quantified using a gas chromatography according to Zhao et al. (2010), (model 663-30, Hitachi Corporation, Tokyo, Japan). The aliquot for the analysis of ammonia-N was measured according to the method described (Russi et al. 2002).

### Statistical analysis

Differences of in vitro measurements were analyzed by in one-way ANOVA procedure (SAS Inc., Cary, NC, USA) version SPSS 19.0 (SPSS Inc., Chicago, IL, USA) to test the differences among treatments. The least square means and standard error of means were presented. Differences among treatments means were separated by Tukey’s test, and the significance level was stated at *P *< 0.05.

## Results

### Ingredients and chemical composition of the experimental diets

The recommended feeding level of RPM depends on the deficiency of the AA in the diet as well as dairy ruminant requirements as defined by the NRC (2001). The present study evaluated the post-ruminal (abomasum–ileum) effects of different supplementation levels of RPM with low protein on digestibility, volatile fatty acids, NH_3_-N, and total tract digestibility using the Rusitec and in vitro techniques. The formulated experimental diets and their chemical compositions are presented in Table 1. The chemical compositions showed that the CP of the HP group was higher than the other groups.

### Rumen-protected methionine supplements their effects on disappearance

Different levels of RPM supplement with low CP diets, their effect on disappearance indexes of the abomasum and ileum were shown in Tables 2, 3 respectively. The disappearance of DM, ether extract (EE), gross energy (GE) and ADF was unaffected in overall treatments (*P* > 0.05) at abomasum and ileum. However, CP disappearance was significantly higher (P < 0.05) in the treatment group (LPLM; LPHM) than HP and parallel (*P* > 0.05) with LP group. Although, NDF disappearance was found significantly (*P* < 0.05) higher in LPHM group than HP but parallel (*P* > 0.05) with LPLM and LP group at both abomasum and ileum.

**Table 2:** Low dietary protein supplemented with rumen-protected methionine effects on disappearance at abomasum using in vitro digestibility technique (means; n = 4)

Substrate disappearance	Treatments				SEM	P value
	HP	LP	LPLM	LPHM		
Dry matter (g/kg DM)	242.92	250.92	252.89	259.55	6.22	0.85
Crude protein (g/kg DM)	517.92^b^	557.18^a^	560.53^a^	566.53^a^	6.45	0.01
Neutral detergent fiber (g/kg DM)	174.10^b^	178.61^a,b^	179.37^a,b^	182.47^a^	1.01	0.03
Acid detergent fiber (g/kg DM)	136.60	129.10	130.31	135.21	2.61	0.73
Gross energy (MJ/kg DM)	314.72	311.22	314.73	315.74	4.79	0.99

*SEM* standard error of the mean

^a,b^ Superscripts values within the same row, are significantly different at (*P *< 0.05)

**Table 3:** Low dietary protein supplemented with rumen-protected methionine effects on disappearance at ileum portion using in vitro digestibility technique (means; n = 4)

Substrate disappearance	Treatments				SEM	*P* value
	HP	LP	LPLM	LPHM		
Dry matter (g/kg DM)	474.41	473.34	476.38	480.87	3.41	0.89
Crude protein (g/kg DM)	774.40^b^	907.58^a^	871.90^a^	875.45^a^	14.49	< 0.01
Neutral detergent fiber (g/kg DM)	400.13^b^	418.75^a,b^	420.41^a,b^	424.08^a^	3.30	0.02
Acid detergent fiber (g/kg DM)	345.44	351.88	358.39	354.44	4.48	0.81
Gross energy (MJ/kg DM)	517.85	507.97	511.44	518.66	3.93	0.77

*SEM* standard error of the mean

^a,b^ Superscripts values within the same row, are significantly different at (*P *< 0.05)

### Rusitec pH and post-ruminal ammonia–nitrogen production

Effects of RPM supplement on Rusitec fermenter pH and post-ruminal ammonia–nitrogen NH_3_-N were presented in Table 4. The pH values before feeding and after feeding from 0, 3, 6, 9, 12, 15, 18, 21 to 24 h of supplemented groups (LPLM and LPHM) were found similar (*P* > 0.05) to LP and HP control groups. Although the supplementation of methionine did not significantly effect on NH_3_-N, the concentration of NH_3_-N production was found non-significant (*P* > 0.05) among supplemented LPLM, LPHM and control HP and LP group at abomasum and ileum.

**Table 4:** Effects of rumen-protected methionine supplementation with low dietary protein on the post-ruminal production of NH_3_-N and Rusitec pH using a rumen simulation technique and in vitro digestibility technique (means; n = 4)

Items	Treatments				SEM	*P* value
	HP	LP	LPLM	LPHM		
pH before feeding^a^	6.60	6.65	6.62	6.64	0.01	0.83
pH after feeding (0–24 h)^a^	6.65	6.68	6.37	6.73	0.01	0.20
Abomasum NH_3_-N (mg/100 mL)	5.72	4.45	4.95	5.78	0.27	0.24
Ileum NH_3_-N (mg/100 mL)	16.27	15.06	15.91	16.23	0.59	0.93

Values within the same row are different at (*P *< 0.05)

*SEM* standard error of the mean

^a^Rusitec pH value

### Effect of supplements with low CP on total and individual volatile fatty acids

The concentration of molar proportions of the daily production of total and individual VFA in the abomasum and ileum are shown in Tables 5 and 6, respectively. No significant differences in the daily production of acetate, propionate, and butyrate were found between LPHM and HP groups; however, significant differences were found between LPLM and LP groups in the abomasum and ileum (*P* < 0.05). The production of total VFA was linearly improved (*P* < 0.05) with LPHM supplementation that were found to be similar (*P* > 0.05) to HP and significantly higher than LPLM and LP groups (*P* < 0.05). However, only the production of valerate in the ileum was found to be the same between the LPHM and HP groups (*P* > 0.05) and was significantly higher than the LPLM and LP groups (*P* < 0.05).

**Table 5:** Low dietary protein with rumen-protected methionine supplementation effects on daily production of total and individual volatile fatty acids using an in vitro abomasum digestibility technique (means; n = 4)

Items	Treatments				SEM	*P* value
	HP	LP	LPLM	LPHM		
Total VFA (mM)	52.81^a^	45.07^b^	48.74^a,b^	51.92^a^	1.23	0.03
Acetate (mM)	29.79^a^	25.34^b^	27.97^a,b^	29.07^a,b^	0.70	0.04
Propionate (mM)	11.8^a^	9.74^b^	10.40^a,b^	11.72^a^	0.36	0.04
Isobutyrate (mM)	0.43	0.41	0.38	0.41	0.01	0.76
Butyrate (mM)	6.76^a^	6.17^b^	6.30^b^	6.64^a^	0.09	0.00
Isovalerate (mM)	1.33	1.47	1.40	1.44	0.04	0.81
Valerate (mM)	2.70	1.94	2.29	2.63	0.13	0.11
Acetate:propionate ratio	2.52	2.60	2.69	2.48	0.03	0.05

*VFA* volatile fatty acids, *SEM* standard error of the mean

^a,b^ Superscripts values within the same row are significantly different at (*P *< 0.05)

**Table 6:** Low dietary protein with rumen-protected methionine supplementation effects on daily production of total and individual volatile fatty acids using an in vitro ileum digestibility technique (means; n = 4)

Items	Treatments				SEM	*P* value
	HP	LP	LPLM	LPHM		
Total VFA (mM)	29.18^a^	23.02^b^	24.44^b^	28.26^a^	0.98	0.00
Acetate (mM)	19.78^a^	16.14^b^	17.20^b^	19.31^a^	0.57	0.00
Propionate (mM)	4.92^a^	4.05^b^	4.29^b^	4.82^a^	0.14	0.00
Isobutyrate (mM)	0.04	0.03	0.03	0.03	0.00	0.24
Butyrate (mM)	2.74^a^	1.77^b^	1.87^b^	2.46^a^	0.15	0.00
Isovalerate (mM)	0.57	0.43	0.45	0.59	0.03	0.11
Valerate (mM)	1.13^a^	0.59^b^	0.60^b^	1.04^a^	0.09	0.00
Acetate:propionate ratio	4.02	3.98	4.00	4.00	0.02	0.96

*VFA* volatile fatty acids, *SEM* standard error of the mean

^a,b^ Superscripts values within the same row are significantly different at (*P *< 0.05)

## Discussion

Mammals are depending on macro and micronutrients for the regulation of physiological functions, health, production, and the prevention of diseases (Abbasi et al. 2014; Stover et al. 2017). Amino acids are natural compounds in many of the aforementioned biological courses (He et al. 2011). Methionine is a sulphur-containing AA that plays a key position in many pathways, i.e. synthesis of phospholipids, carnitine, creatine, polyamines, phosphatidylcholine, precursor of succinyl-CoA, homocysteine, cellular methylation (Soares et al. 2017), deliver sulphur groups for the synthesis of cysteine and reduce dietary cysteine requirement (Mackay et al. 2012). The present study was designed to measure which concentration of RPM with low dietary CP effects maximum post-ruminal, to help for further application in vivo study. For maximum VFA production, disappearance and essential AA at ileum to maintain dairy performance and solve environmental problems linked to high protein feeding in ruminant’s industry.

In the current study, RPM supplementation significantly promotes disappearance and fermentation of diets, as the concentration increases from 0.11 and 0.81 g/kg (DM basis). The post-ruminal disappearance of CP and aNDF were significantly higher in LPLM and LPHM, but no effects were found on DM, ADF, and GE with RPM supplementation. Like our current findings, previously reported that dry matter intake averaged 25.4 kg/day was not affected by RPM treatment (Zang et al. 2006). Consistent with these results, in other trials it was observed no DMI improvement through supplementing RPM (Lee et al. 2015). A DMI response to RPAA supplementation was recorded negligible in studies with early-lactation cows (Lara et al. 2006). Conversely, Lodman et al. (1990) recorded a numerical improvement in the extent and rate of DM and NDF digestion, because of urea and methionine supplementation. The post-ruminal production of total VFA was improved significantly, particularly, SCFAs, (acetate, propionate, and butyrate) were significantly improved by supplementation of RPM. Our findings support earlier observations that, the SCFAs, primarily acetate, propionate, and butyrate, are organic acids produced in the intestinal lumen by bacterial fermentation of mainly undigested dietary carbohydrates, specifically resistant starch and dietary fiber and, to a lesser extent, dietary and endogenous proteins (Fan et al. 2015). However, it was noted that propionate originating from fermentation is the major precursor of glucose in cows which contributes up to 60% of glucose flux rate (Larsen and Kristensen 2013). For ruminants, glucose is the important source of milk lactose synthesis. It is mostly provided by the liver (up to 90%) through gluconeogenesis process (Nafikov and Beitz 2007). Furthermore, Mulligan et al. (2002) stated that both total VFA and synthesis of acetate associated with a NDF degradability. However, in current study, at ileum the production of valerate was significantly increased with high supplementation of RPM, the results was similar to previous study of Mackie et al. (1991), who reported that, with supplementation of DL-Met the significant increase in iso-valerate and valerate observed which contributed to a greater total VFA, because of branched-chain VFA (Leu, Ile, Val) originates from the bacterial deamination moreover, greater branched-chain VFA indicates that methionine analog supplements increased the hydrolysis of protein. The pH plays a significant role to maintain normal homeostasis of the gastrointestinal tract. Maximum and balance pH is important for normal digestibility of experimental diets in simulation apparatus. The NH_3_-N concentration in the Rusitec effluent depends on the degradation and N uptake efficiency by post ruminal microorganism, and production of NH_3_-N play the main role in MCP synthesis (Bach et al. 2005). In the present study, the post-ruminal concentration of NH_3_-N was unaffected significantly by RPM supplementation compared to the control groups. However, previous studies indicating that ammonia absorption (0.9 g/day with the silage diet) occurred between the rumen and duodenum and probably from the omasum (Engelhardt and Hauffe 1975). Ammonia that entered the ileum was mostly absorbed before the digesta reached the ileum (Siddons et al. 1985). Results indicated that RPM supplementation did not significantly alter the Rusitec fermenter pH before changing experimental feed or after changing from 0 to 24 h compared to the control group. The result agreed with Papas et al. (1984) who reported that, rumen-protected methionine was 94% stable in acetate buffer pH 5.4 in the rumen, however, polymeric coatings are usually pH-sensitive and intended to maintain their structural integrity at the pH normally encountered in the rumen, furthermore, in citrate buffer pH 2.9, which simulates the abomasal environment, and 90% of methionine was released. The lower pH of the post ruminal (abomasum and ileum) activates the breakdown of the coating and consenting release of amino acids for absorption. Furthermore, the current finding supported by Silva et al. (2016) who reported that dietary CP levels not altered ruminal pH in finishing beef cattle. The discrepancy between the in vivo and in vitro three-step procedure estimates may partly be due to the protein digestion, RPM supplementation and post-ruminal effects (Calsamiglia and Stern 1995). Moreover, another cause of the discrepancy between the in vivo and in vitro three-step procedure might be the incubation time in the Rusitec.

In conclusion, different levels of ethyl-cellulose RPM supplemented with low CP and their post-ruminal effects showed that the LPHM group promoted disappearance, improved fermentation, increased post-ruminal fiber degradation, and promoted the total tract digestibility of nutrients. Future research should proceed in vivo to address the mechanism involved in producing such effects by RPM supplementation and the way that oxidative status might affect subsequent reproductive and productive performance of dairy ruminants.
